# Long-term maintenance therapy with ketamine infusions for treatment-resistant depression: a retrospective chart review

**DOI:** 10.1192/j.eurpsy.2026.10592

**Published:** 2026-07-31

**Authors:** E. Peters, A. Al Hasani, K. Shukla, K. Halpape

**Affiliations:** 1 Psychiatry, University of Saskatchewan, Saskatoon; 2 Saskatchewan Health Authority, Regina; 3 Pharmacy and Nutrition, University of Saskatchewan, Saskatoon, Canada

## Abstract

**Introduction:**

Ketamine therapy for treatment-resistant depression has a well-established rapid antidepressant effect for acute symptom management. However, there is far less research on the efficacy of long-term maintenance therapy for relapse prevention.

**Objectives:**

To examine the utility of long-term maintenance therapy with ketamine infusions delivered in a naturalistic treatment setting.

**Methods:**

We conducted a retrospective chart review of data from 56 outpatients with treatment-resistant depression receiving intravenous ketamine therapy in Saskatchewan, Canada. Depressive symptoms were assessed before each treatment session with the 17-item Hamilton Depression Rating Scale (HAM-D). The lowest HAM-D score after 3-6 infusions marked the start of maintenance therapy. From this point forward, Kaplan-Meier survival curves were used to estimate the probability of relapse (i.e., HAM-D score increase ≥ 25%, for two consecutive visits) over the next 365 days.

**Results:**

Patients received, on average, 14 maintenance infusions over 253 days. The average number of days between infusions, per patient, was 25.5 (*SD* = 8.86). Average HAM-D scores at the start of maintenance (*M* = 17.2, *SD* = 7.31) did not increase by the final session (*M* = 16.5, *SD* = 8.81; *t* = -0.94, *p* = .35). After 365 days, the estimated relapse rate was 21-36% depending on the minimum HAM-D score required to define a relapse. The average time to the start of a relapse was approximately 6 months, but individual times ranged from 14 to 328 days.

**Conclusions:**

Long-term maintenance therapy with intravenous ketamine can be continued for up to a year alongside conventional treatment. During this time, roughly a third of patients may experience a persistent depressive symptom exacerbation of unknown clinical significance. More research is needed to determine the nature of these symptom exacerbations.

**Disclosure of Interest:**

None Declared

